# Killing Me Softly—Future Challenges in Apoptosis Research

**DOI:** 10.3390/ijms15033746

**Published:** 2014-03-03

**Authors:** Mike-Andrew Westhoff, Oliver Brühl, Lisa Nonnenmacher, Georg Karpel-Massler, Klaus-Michael Debatin

**Affiliations:** 1 Department of Pediatrics and Adolescent Medicine, University Medical Center Ulm, Ulm 89075, Germany; E-Mails: andrew.westhoff@uniklinik-ulm.de (M.-A.W.); lisa.nonnenmacher@uniklinik-ulm.de (L.N.); 2 Laboratorio Analisi Sicilia Catania, Lentini (SR) 96016, Italy; E-Mail: kontakt@oliverbruehl.de; 3 Department of Neurosurgery, University Medical Center Ulm, Ulm 89081, Germany; E-Mail: georg.karpel@uniklinik-ulm.de

**Keywords:** apoptosis, cancer therapy, microenvironment, combination therapy, chronification

## Abstract

The induction of apoptosis, a highly regulated and clearly defined mode of cell dying, is a vital tenet of modern cancer therapy. In this review we focus on three aspects of apoptosis research which we believe are the most crucial and most exciting areas currently investigated and that will need to be better understood in order to enhance the efficacy of therapeutic measures. First, we discuss which target to select for cancer therapy and argue that not the cancer cell as such, but its interaction with the microenvironment is a more promising and genetically stable site of attack. Second, the complexity of combination therapy is elucidated using the PI3-K-mediated signaling network as a specific example. Here we show that the current clinical approach to sensitize malignancies to apoptosis by maximal, prolonged inhibition of so-called survival pathways can actually be counter productive. Third, we propose that under certain conditions which will need to be clearly defined in future, chronification of a tumor might be preferable to the attempt at a cure. Finally, we discuss further problems with utilizing apoptosis induction in cancer therapy and propose a novel potential therapeutic approach that combines the previously discussed features.

## 1. Introduction

**Figure 1.**
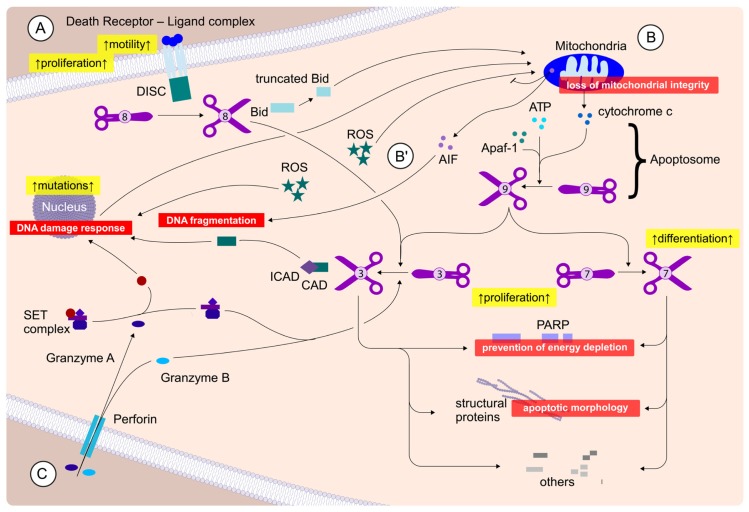
Apoptosis induction and signaling. Apoptosis can be initiated via three distinct routes: (**A**) Extrinsic pathway: Ligands bind to death receptors leading to formation of the DISC complex and activation of caspase 8 (depicted as scissors). Caspase 8 either directly activates, via cleavage, caspases 3 and 7, or needs to enhance the “death signal” via Bid cleavage which thus initiates a mitochondrial feedback loop. Whether cells depend on this mitochondrial enhancement is traditionally determined experimentally and dying cells are either referred to as type I (no mitochondrial amplification necessary) or type II (mitochondrial amplification necessary) cells; (**B**) Intrinsic pathway: Chemotherapy and radiation, among others, cause DNA damage either directly or by producing oxygen radicals (ROS), which can either further impair the nuclear DNA integrity or compromise the mitochondria. This leads to the activation of mitochondrial death signaling, via the release of cytochrome c and other factors into the cytosol. Here, cytochrome c combines with caspase 9, Apaf-1 and ATP to form the Apoptosome which activates caspases 3 and 7. Of note, there is also a caspase-independent form of apoptosis (**B**′), whereby AIF, which can also function as a ROS scavenger, gets released from the mitochondria and triggers chromatin condensation and DNA fragmentation; (**C**) CTL (Cytotoxic T lymphocytes), or NK (natural killer) cell-mediated apoptosis: A specialized form of apoptosis whereby the immune system triggers apoptosis by inserting a perforin “tunnel” in its target cell. This leads to granzymes entering the cell and activating caspase 3 and 7. All forms of caspase-dependent apoptosis converge at the level of caspase 3 and 7, the executioner caspases, which then proceed to cause the structural alterations to the cell, either directly or via activation of CAD DNase. Typical cellular responses to apoptosis are depicted in red, while in yellow effects of pathway activation are shown, which are undesirable during cancer therapy.

Despite the emergence of alternative paths leading to cell death, such as necroptosis [1] and—more controversially—autophagy [2], apoptosis remains, due to its limited side effects, the major preferred mode of action to eliminate the tumor burden of cancer patients. While we habitually refer to apoptosis as “programmed cell death” or “suicide”, a more correct, although less salient term would be “energy-consuming process that regulates cell dying”, as in terms of therapeutic implications it is more advantageous to think of it as a process initiated by death signals (may they be drugs, radiation or proteins), rather than as the mere consequence of poisoning. Cell dying, from the induction of DNA damage or death receptor activation when the cellular remains are taken up by macrophages [3], can take anywhere from several hours to many days. It is during that period when the most interesting events occur within the cell and when the unique features of cancer counteract and prevent the death signal. Interestingly, Nixon’s “war on cancer” which aimed to find a cure for these malignancies within the next 25 years was declared the year prior to the publication of Kerr, Wyllie and Currie’s seminal paper which first defined apoptosis [4]. In the intervening 40-odd years we have reached a near comprehensive mechanistic understanding of apoptosis (summarized in Figure 1), however the therapeutic successes on the whole have been less forthcoming.

Two of the three pillars of modern cancer therapy, radio- and chemotherapy, work predominately by inducing apoptosis, the third—surgical resection—is, cases of pre-metastatic cancer aside, seldom fully effective if not augmented by the other two. Apoptosis is a naturally occurring mechanism that plays a vital role during development, immune system priming and in the maintenance of tissue homeostasis [3,5]. The appearance of discontinuous uncontrolled growth, *i.e.*, cancer, means that homeostasis is no longer maintained and a sub-population of cells, which we can now refer to as “malignant”, has escaped the body’s natural surveillance, either by genetic or epigenetic means. This apoptosis resistance is one of the defining hallmarks of cancer [6]. Therapeutic intervention basically tries to restart this intrinsic feature of cells, namely to commit suicide when they become abnormal, but, of course, in cells that have previously already escaped this signal. The consequence is that high doses of radiation or chemotoxic substances are needed to overcome the block in apoptosis, leading to the collateral damage of healthy tissue. To maximize apoptosis of malignant cells, while concurrently minimizing the damage sustained by normal cells, one should consider alternate focuses of attack. We propose three such approaches in this review: (1) only indirectly target the highly resistant cancerous cells and alternatively aim at the tumor’s interaction with its microenvironment; (2) Augment traditional therapeutic approaches with carefully timed additions of pharmacological signaling inhibitors or therapeutic antibodies; (3) Consider an alternative primary aim as desired therapeutic outcome, enhanced and prolonged quality of life versus tumor-free survival.

## 2. Discussion

### 2.1. AMARe Mortis

**Figure 2.**
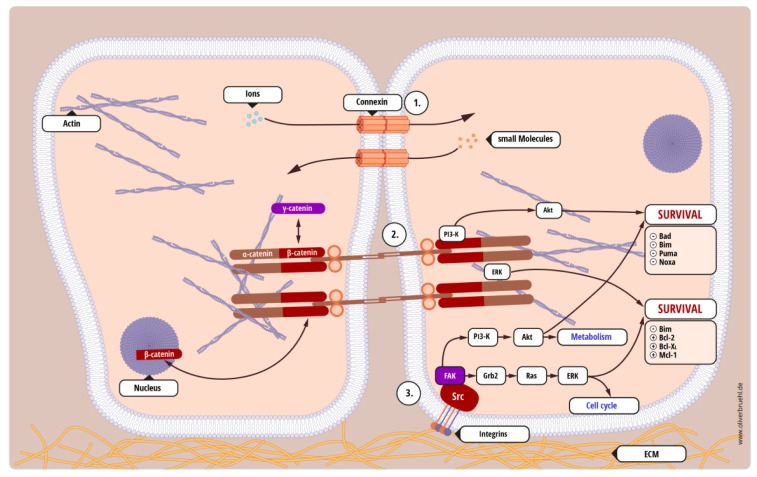
The tumor’s interaction with its solid microenvironment. Three of the five common forms of adhesions are depicted here and their role in cell death prevention is highlighted (expanded from [40]): (**1**) Gap junctions, aqueous channels consisting of connexins, through which ions and small molecules can be exchanged between adjacent cells. Generally, they are considered more in terms of cell-cell communication rather than cell-cell adhesion, however, we previously showed both that gap junctions contribute to glioblastoma cell-cell adhesion and, by diluting the apoptotic signal, reduce cell death [28]; (**2**) Adherens junctions are not only contacts between cells, but also anchorage points to the cytoskeleton. They consist of plasma membrane spanning cadherins and adjacent cadherin interactions on the plasma membrane lead to the formation of a zipper-like structure which provides a strong adhesive link between cells. In addition, components of the adherens junctions not only mediate cell-cell adhesions, but are also involved in the regulation of signaling cascades, in particular the *Wnt* pathway; (**3**) Focal adhesions are links between the extracellular matrix and the actin cytoskeleton. Their dynamic regulation plays an important role in cell motility, while there are also sites of origin for (a) intracellular survival signals and (b) extracellular cues for microenvironment remodeling. As example, we show in detail two survival pathways that are activated by adherens junctions and focal adhesions, the Raf/MEK/ERK pathway and the PI3-K/Akt signaling cascade (see Figure 3). Note that (−) indicates reduced, while (+) stands for increased protein activity. Not shown are desmosomes, which act as tethering points for intermediate filaments, and tight junctions, which form a boundary between apical and basal domains of the plasma membrane. While the former are usually exclusively discussed in terms of reduced expression during metastasis [45], the role of tight junctions is more controversial, as its components either found to be up- or down-regulated in cancers [46]. Of note, PTEN, the negative regulator of PI3-K/Akt signaling also regulates tight junctions [47].

One of the most obvious updates that occurred between the 2000 and 2011 version of Hanahan and Weinberg’s seminal work on the hallmarks of cancer [6,7] is that in the latter the role of non-tumorgenic factors is much more emphasized. No longer is a cancerous growth purely understood in terms of transformed cells only, but the direct surroundings, the so-called microenvironment, is also taken into account. We feel this is an important, long overdue shift in focus, as studies already from the same time period as the earliest apoptosis studies [8,9] indicated that the cellular interaction with its (solid) microenvironment might affect the tumor cell’s ability to survive treatment. Importantly, the limitations of *in vivo* culture systems, *i.e.*, cell culture in plastic flasks, which lack a physiological microenvironment were also already noted and a “spheroid” model of cell culture to—at least partially—counter the *in vitro* limitations was developed [10]. However, till recently the role of the microenvironment in cancer was mainly investigated in the context of metastasis and invasion, in particular since anoikis, a specialized form of apoptosis which occurs upon the loss of cell-matrix adhesion, is often seen as restricted to non-transformed cells [11]. It is only relative recently that the concepts of CAM-DR [12–37], cell-adhesion mediated drug resistance, and—probably still to a lesser extent—CAM-RR [9,10,12,38,39], cell-adhesion mediated radiation resistance, have gained interest in the scientific community. There are several different types of interaction (Figure 2), which we previously categorized into three groups [40]:

1. Homotypic cell-cell interaction—tumor cells interact with other adjacent tumor cells;
2. Heterotypic cell-cell interaction—tumor cells interact with other non-malignant cells, for example tumor-associated stroma or healthy tissue that is being invaded;
3. Cell-substrate interaction—tumor cells interact with solid structures, such as the extracellular matrix or the basal membrane.

While restoration of the “normal” unaltered microenvironment can be sufficient to block tumor progression and tumor formation [41,42], it is important to note that these adhesions do not primarily mediate cellular survival. Their absence does not necessarily lead to anoikis, as anchorage-independent growth is also a hallmark of cancer [6], but they form communication points between cells or sites from which pro-survival signals can be mediated upon stress. For example, the presence of the extracellular matrix, through cell-substrate interactions, not only facilitates cellular locomotion and thus invasion [43], but during migration it also enhances the cells’ resistance to apoptosis induction [44].

Furthermore, our own work in glioblastoma has also shown that differences in the microenvironment can significantly alter cellular behavior and—maybe even more importantly—that glioblastoma cells themselves alter their environment depending on their position within the tumor [28,49]. We previously showed that glioblastoma cells have developed different modes of interaction with their microenvironment and that by inhibiting one form of interaction, *i.e.*, blocking cell substrate adhesion, we did not sensitize cells for apoptosis but only drove them to enhanced (gap junction-mediated) cell-cell interactions [28]. Importantly, only by blocking both types of interaction, could we sensitize these cells for therapy-induced apoptosis [28]. Proceeding from this work, we recently were able to add additional pieces to the puzzle by showing that both, *in vitro* and *in vivo*, glioblastoma cells alter their microenvironment by secreting fibronectin, in essence creating their own extracellular matrix [49]. Interestingly, *in vivo*, only the cells either at the invading edge of the tumor or those that have already migrated away from the tumor bulk produced fibronectin, while those within the tumor presumably interact via gap-junctions [49]. Blocking cell-substrate interactions led to reduced invasion and tumor shrinkage [49]. We are currently examining the possibility of blocking, both cell-cell and cell-substrate interactions, thus extending the therapeutic window and sensitizing cells to apoptosis.

This is not an isolated phenomenon; expanding from our previous report, Table 1 lists twenty-seven occurrences of AMAR (adhesion-mediated apoptosis resistance [40]) in a wide range of cancers and identifies the molecular mechanisms if known. It is striking that the molecules involved in any survival strategies mediated by AMAR are identical to those currently being targeted via pharmaceutical inhibitors or therapeutic antibodies, such as the PI3-K signaling cascade or the Bcl-2 family (highlighted in Figure 2). However, why do we believe targeting the interaction of the cancer cell with its microenvironment is more promising than targeting the cancer cell directly? Cancer cells within a population are almost by definition a highly unstable target with high mutation rates [53], thus the risk of allowing a fast proliferating population to escape treatment restrains by genotypic or, indeed, epigenetic means is always high. In contrast, the non-tumorigenic microenvironment is a much more stable target. Indeed, the reversion of tumor-associated cells of the microenvironment back to a normal phenotype has been shown to be readily achieved [54] and Table 1 clearly demonstrates that, aside from the aforementioned possibility where this approach is sufficient to block tumor progression [42], it leads to an apoptosis sensitization in a vast array of tumors. Also as the interaction of tumor cells with their microenvironment is initiated at the cell surface, it is a relative easy target, as pharmacological inhibitors and therapeutic antibodies do not need to be taken up by the cells. Finally, while apoptosis resistance can be mediated almost at any given position within the apoptosis cascade, or by hyperactivation of survival signals, there is a much more limited range of often closely related surface molecules, *i.e.*, less potential targets and less molecules that can compensate for any protein successfully targeted.

**Figure 3.**
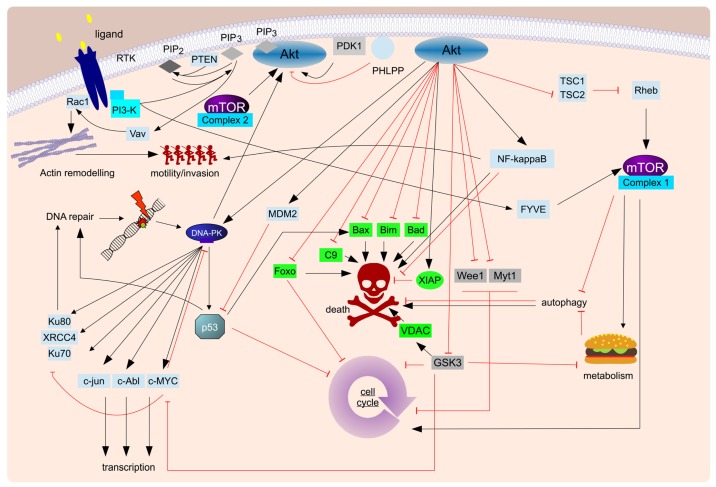
The so-called PI3-K/Akt/mTOR survival pathway. The complex signaling cascades initiated by the near-apical PI3-K are usually referred to as the PI3-K/Akt/mTOR survival pathway. Shown here is a highly simplified diagram of the non-controversial interactions that have been described in the literature, with particular emphasis on the potential therapeutic targets within this signaling complex, PI3-K, mTOR, Akt and DNA-PK, while the pro-apoptic molecules that can be blocked by activation of the PI3-K/Akt/mTOR pathway are highlighted in green. Of interest are two particular aspects: (**1**) The complexity of interactions which—depending on cellular context, outside influences and time points investigated—can lead, in extreme cases, to diametrically opposite outcomes. For the sake of clarity the interaction and crosstalk with other equally complex signaling cascades, most notably MAP kinase signaling [48], is not shown; (**2**) While cellular survival is indisputably a key aspect regulated by PI3-K, other cellular aspects of equal interest to cancer research are also affected by PI3-K signaling, such as motility, metabolism, cell cycle progression and DNA damage repair (underscored). Considering both of these points it becomes apparent that effective modulation of this cascade in order to enhance therapeutic efficacy cannot be simply reduced to a strong, prolonged inhibition of the most apical proteins.

There are two major caveats frequently expressed with regards to this strategy. First, reducing cellular adherence might enhance metastasis. While obviously more work in this area needs to be done, it is worth bearing in mind, that metastasis is the cumulative effect of increased motility and invasion of the surrounding tissue. Motility is not achieved by reduced adhesion to surrounding cells, but by dynamic regulation of those adhesions. Indeed we could show for glioblastoma cells that less adhesive cells are actually less invasive [49]. Indeed it has even been suggested that under certain circumstances microenvironmental changes at a future site of metastasis precede the establishing of secondary tumors. This formation of a premetastatic niche [55,56] is mediated by a process called dynamic reciprocity and blocking the environmental interaction of cancer cells and niche should reduce metastatic spread and increase the likelihood of circulating tumor cells to undergo anoikis. Therefore, one of the side effects of targeting the tumor cells’ adhesions can actually be a reduction in size and distance from tumor bulk of metastasis [49], a more localized disease and therefore an increase of the therapeutic window; Second, it might provide a far less comfortable therapeutic window than targeting survival signals within the cancer cell to which it is inferred to be addicted. However, it should be pointed out that there have been undisputed successes in targeting the interaction of tumor cells with their surroundings (summarized in Table 1) and that several survival signals that are enhanced by AMAR are also considered key signaling cascades in cancer progression, such as, for example, PI3-K mediated signaling (Figures 2 and 3). Furthermore, the aforementioned dynamic reciprocity, which is best understood as the continuous bidirectional interaction between tumor cells and their surrounding cellular and extracellular microenvironment [57] also needs to be considered here. The microenvironment not only provides an anchorage point for genetically unstable cancer cells that mediates AMAR, signals from the tumor cells also alter the microenvironment turning it into a specialized tissue distinct from healthy, “normal” tissue without influencing its genetic make-up.

In summary, using one of the most enduring cancer models, Paget’s “Soil and Seed” hypothesis [58], we propose to target the soil and not the seed, thus more effectively preventing cancer dissemination and propagation.

### 2.2. Tempus Occidendi et Tempus Sanandi

Critics often unfairly refer to the current cancer treatments collectively as “cut, poison and burn” (see, for example, http://cutpoisonburn.com), there is—due to the very nature of what cancer is—a grain of truth in that statement: Successful cancer therapy will always be a balancing act between killing as many cancer cells as possible on the one hand and minimizing the side effects for the patients’ healthy tissue on the other. Aside from rare exceptions where we have a target specific to cancer, for example in the case of novel fusion proteins such as BCR-ABL [59], anything that induces apoptosis in cancer cells will also potentially induce apoptosis in healthy cells. The current trend in treatment of cancer is moving away from the so-called high dose density paradigm, administering chemotherapeutic doses at or near the limit of tolerable doses, towards the metronomic therapy [60]. The latter approach administers lower doses of drugs continuously or at frequent intervals, to reduce cytotoxic side-effects and increase treatment efficacy [61]. A strategy currently employed to tilt the scales in the patients’ favor—that is to say to further reduce the side-effects, while maintaining efficacy against the tumor—is to minimize the cancer cells’ resistance towards therapy, in essence sensitizing the tumor for apoptosis induction, allowing equal or better outcome with less side-effects. This approach is based on the alterations in signaling pathways that lead to the specific hallmarks of cancer [6].

The more essential such a pathway is and the more commonly it is altered in different cancers, the more promising it is as a potential co-target in therapy. One of the most frequently activated signaling cascade in cancer is the PI3-K/Akt/mTOR pathway (Figure 3) [62], which is not only involved in cell survival and invasion [63], but also in proliferation, DNA damage repair and cell growth/metabolic regulation [64,65]. The PTEN gene, the gene product of which is a negative regulator of PI3-K/Akt/mTOR signaling, and PIK3CA, which encodes a catalytic subunit of PI3-K, are frequently mutated in, depending on the specific cancer, around a third of all tumors [66], loss of PTEN heterozygozity is described in 59% and 76% of glioblastoma and uveal melanoma, respectively [67]. Indeed, it is often cited as the second most frequent mutated gene in cancer (for example [68]). There is also compelling preclinical evidence that inhibition of this pathway restores sensitivity to therapy in breast cancer, non-small-cell lung cancer and glioblastoma [69], while additional cancer entities, such as renal cell carcinoma, neuroblastoma, pancreatic neuroendocrine tumors and leukemia [70–73] are currently being further evaluated. At least twenty pharmacological inhibitors targeting various (isoform-) specific targets of this signaling cascade are currently at various stages of development [67]. Several clinical trials, phase I–III, suggest that most inhibitors of PI3-K are well-tolerated and have little side effects [69,74].

Interestingly, the current clinical paradigm regarding combination therapy seems to use a constant high level of a pharmacological inhibitor followed by addition of conventional chemotherapy, based on the assumption that first the survival signal has to be blocked than the drug can exert a more potent effect. However, recently, we and others have shown that timing and length of inhibition play a crucial and occasionally counter-intuitive role [75,76]. Lee and colleagues showed that maximal sensitization to doxorubicin-induced apoptosis could be achieved for triple-negative breast cancer cells by 24 h (but not 48 h) prestimulation with elotinib [75]. This sensitizing effect was not seen during co- or poststimulation with elotinib and was independent of cell cycle progression/proliferation and initial DNA damage and “dynamic rewiring of oncogenic signaling” has been proposed as cause [75]. Our own work with doxorubicin independently confirmed a similar effect in neuroblastoma (and to a limited extent glioblastoma also): Here we could show that posttreatment with NVP-BEZ235, a dual PI3-K/mTOR inhibitor, sensitizes cancer cells for doxorubicin-induced apoptosis to a far greater extent than co- or pretreatment [76]. Interestingly, pretreatment even desensitizes neuroblastoma cells for doxorubicin-induced cell death, which has lead us to propose the following model: The exposure to doxorubicin probably induces death signaling at several different sites within the cell, all of which cumulate at the mitochondria to induce cell death via the intrinsic apoptosis pathway. This death signal is not transduced further, unless inhibition of PI3-K-activated Akt leads, via GSK-3β, to Porin modulation and loss of mitochondrial membrane potential, cytochrome c release and caspase activation [76]. Therefore the role of PI3-K-mediated signaling in the context of neuroblastoma cell survival after doxorubicin exposure is at the level of mitochondria. Prolonged exposure to the inhibitor used also leads to a cell cycle arrest and the induction of autophagy [76], in essence altering the metabolism of the cell, changing the energy priorities and therefore the role of the mitochondria. Optimal sensitization is only achieved, when PI3-K-dependent signaling at the mitochondria is inhibited, but prior to metabolic alterations in the cells that occur as a consequence of prolonged inhibition of the PI3-K/Akt/mTOR network. The future challenges will lie in how to translate these findings [75,76] into a clinically relevant setting. Furthermore, algorithms and databases need to be developed to understand how each combination, which in future will be likely to grow more complex than the one inducer, one sensitizer combinations currently investigated, affects which tumor (sub)type under which conditions. This is a daunting task, but computational power and high throughput assays have finally reached a stage where it becomes a feasible and, in our opinion, promising undertaking.

In conclusion, we believe it is wrong to view most pathways as linear entities with one defined function, *i.e.*, “the PI3-K/Akt/mTOR survival pathway”. These signaling cascades are networks with multiple often independent functions. To further improve combination therapy we need to understand not only which molecules we wish to target within a pathway, but when and for how long to target them to optimize the sensitizing effect. Furthermore, we must be aware that optimal sensitization for apoptosis is not necessarily equivalent to maximal inhibition of the targeted “survival pathway”.

### 2.3. Caedite eos?

**Figure 4.**
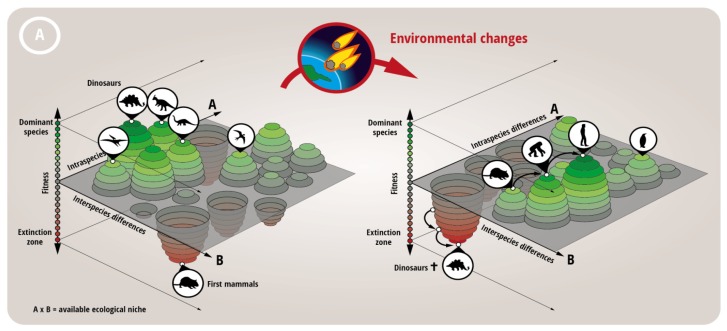

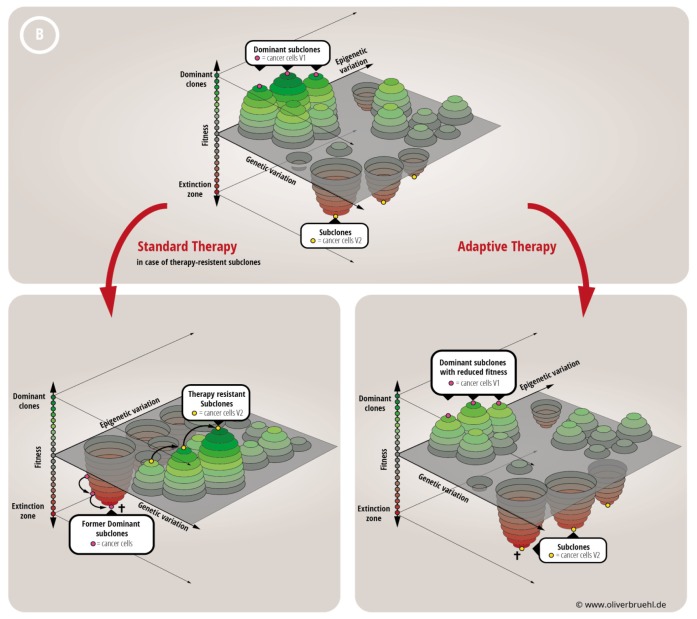
Tumor cell population dynamics and the potential of chronifying treatment strategies. In recent years the subclonal and microenvironment-dependent composition of tumor populations has become apparent. However, lessons learned from population genetics and ecology have only just begun to be applied to the design of novel treatment strategies (for example [81,82,84,85]). (**A**) Probably the best known example of what can happen to species (simplified here as various dinosaurs and a proto-mammal) competing for a single ecological niche (Earth) upon a drastic change in the environment (a comet hitting the planet) is the extinction of the dinosaurs and rise of the mammals during Cretaceous-Paleogene extinction event (better known as K-T extinction). The alterations in the environment (which include the absence of the hitherto dominant species) allow a minor species to repopulate the niche and evolve to new dominance; (**B**) The dynamics described above can also be recapitulated for the ecological niche in which a tumor grows. Therapy causes drastic alterations in the tumor microenvironment (which include the absence of the hitherto dominant subclone), which can allow a minor, therapy-resistant subclone to repopulate the niche and evolve to new dominance (lower left). Traditionally alternative therapeutic approaches or dose escalation are then attempted to control the malignancy. An alternative approach, as suggested by [83] and shown in the lower right, is to reduce the overall fitness of the tumor by reducing the fitness of the dominant clone, but keep the dominance intact. This can result in a stable microenvironment and a disease stasis [83].

Traditionally, the medical profession has always seen it as their aim to heal the patient. However, one has to consider whether this approach maximizes the patients’ chances for a long and unaffected life. As we have alluded to in Section 1, a recent trend is emerging using population genetics, and in essence treats cancerous tumors as fast evolving populations in a relatively stable environment (the human body, the microenvironment, see above). This trend is fairly new and still in its infancy, as data on the composition of tumors, the presence of subclones and the extreme heterogeneity, only now become available as large scale sequencing approaches become more common. However the strong parallels between a given population of organisms interacting with an environment and cancer cells with the tumor environment/microenvironment are hard to ignore: Any successful attempt at treating the malignancy is an environmental chance that leads to evolutionary pressure, natural selection and the emergence of a new population that is better adapted to the novel environmental conditions, or in essence a therapy-resistant subclone becomes the dominant clone. Importantly, current data indicate that said subclones are actually often already present at diagnosis, within a given tumor a large cellular heterogenity exists, reflected in altered protein expression [77]. These subpopulations are kept in check by other better adapted populations of cancer cells [78,79]. This means that the fitness landscape of a tumor is to be considered fairly rugged. Combined with recent data on the additional epigenetic landscape Huang, for example, concludes “Darwinian selection (within a tumor) is not operating at maximum efficacy” [80]. If the tumor responds to treatment, basically the induction of selective pressure, this induces an extinction event and cancer relapse is then dependent on a combination of initial population size and mutational—and presumably also epigenetic [81]—fitness distribution [82]. Basically the dynamic mechanisms at play that led to the extinction of the dinosaurs and the rise of our mammalian ancestors (who were competing for similar ecological niches) are the same that lead to the emergence of a treatment-resistant tumor after massive apoptosis induction (Figure 4). Based on this model a more flexible approach, adaptive therapy, has been proposed [83]: Using the relatively crude measurement of tumor size as determinant, the authors of this study compared an adaptive therapy, where the drug is only given when tumor size surpasses a critical volume, with a standard fixed regime. The adaptive therapy led to an overall reduced tumor burden and a prolonged chemosensitivity of the malignancy [83]. Further analysis of these findings confirm the above described model, the fitter, chemosensitive cells (*i.e.*, the dominant subclone) suppress the growth of the less fit, but resistant cells [83]. Additional, surprising findings of these experiments have so far not been explained and need further investigation, in particular that the author needed progressively lower drug doses and longer time intervals between administrations to achieve tumor growth control. The authors of the above quoted study will no doubt be the first to acknowledge that the adaptive study performed is rather limited, tumor size is an unreliable readout that, for example, ignores the problem of metastasis, and a truly adaptive study would have used different drugs with different mechanisms of action. Nevertheless the findings are a potent proof of principle that indicates that therapies with an alternative end point, not focused on using the maximal tolerated dose of therapy to fully eradicate the tumor, can be in the long run more beneficial for the patient.

These ideas can also be successfully translated into a clinical setting, as our recent work on the so-called RIST therapy demonstrates [86]. We found that a therapy consisting of inhibitors targeting aberrant signaling in cancer cells (Rapamycin and Sunitinib) combined with chemotherapy (Irinotecan and Temozolomide) enhances apoptosis and reduces proliferation of glioblastoma cells compared to single agents alone, both *in vitro* and *in vivo* [86]. Importantly, as part of an experimental approach in compassionate use setting, meaning only where all convenient treatment options had already failed, children with recurrent solid tumors, particularly with glioblastoma, were treated with the RIST therapy, a metronomically designed protocol. Overall, we found that for many patients survival time was prolonged, but the tumor burden could not be completely diminished. Two index patients with glioblastoma were treated with RIST therapy after surgical resection and treatment with temozolomide and irradiation resulting in a stable tumor burden over a long period of time, up to 66 month [86]. After cessation of treatment tumor progress was noted, but, importantly, after resumption of therapy a stable state could be regained.

Interestingly, at least for a subgroup of cancers that mediate their treatment resistance not by genetic but epigenetic means (see for example [87,88], as well as our own work [89]), this approach could be particularly advantageous. Recent modeling suggests that populations consisting of individuals with high phenotypic plasticity, often exhibit—in the context of a multipeaked fitness landscape—an enhanced evolutionary rate, but at the price of decreased average fitness [90]. If these findings can indeed be translated into a tumor setting, this might indicate that tumors that quickly develop resistance to a given therapy are also the tumors which are best suited to a chronification approach.

## 3. Conclusions

While apoptosis is still considered the preferable mode of killing cancer cells, as healthy tissue and therefore the whole patient are less negatively affected by it than by necrosis [102], recent data also suggest an undesirable effect on surviving tumor cells [103]. A “Phoenix Rising” pathway has been proposed, whereby classical caspase-dependent apoptosis induces potent growth-stimulating signals that can induce repopulation of the tumor via Prostaglandin E2 secretion [103]. Indeed, the unsuccessful induction of apoptosis can also have several undesirable side effects, such as increased caspase-mediated tumor cell migration and metastasis [104,105], or induction of therapy resistance via damage to the tumor microenvironment [106]. In addition caspase activity has also been shown to play a key role in differentiation of stem cells [107,108] and it remains to be elucidated how this affects tumor progression. In summary, there are several aspects concerning apoptosis outside the scope of this review that need to be addressed to further improve therapeutic outcome. Nevertheless, should the points and hypotheses raised here be validated in a large-scale clinical setting, we would propose following an approach to enhance apoptosis induction during cancer therapy and thus extend the patients’ quality and quantity of life.

Inhibit cancer cell/microenvironment interaction and adhesion, thus priming the cells for apoptosis while concurrently extending the therapeutic window by blocking invasion and metastasis. Then combine conventional therapy (surgery, radio- and chemotherapy) with pharmaceutical inhibitors and therapeutic antibodies in the sense of a complex combination therapy to reduce the tumor burden. Finally, and there we see the greatest need for an open and frank debate regarding the ethical consequences, decide whether a maximal reduction in tumor burden, *i.e.*, a cure, should be attempted, or whether a chronification of the malignancy, *i.e.*, keeping tumor size constant at a level that minimizes the growth’s effect on the patient, would be more desirable.

**Figure 5.**
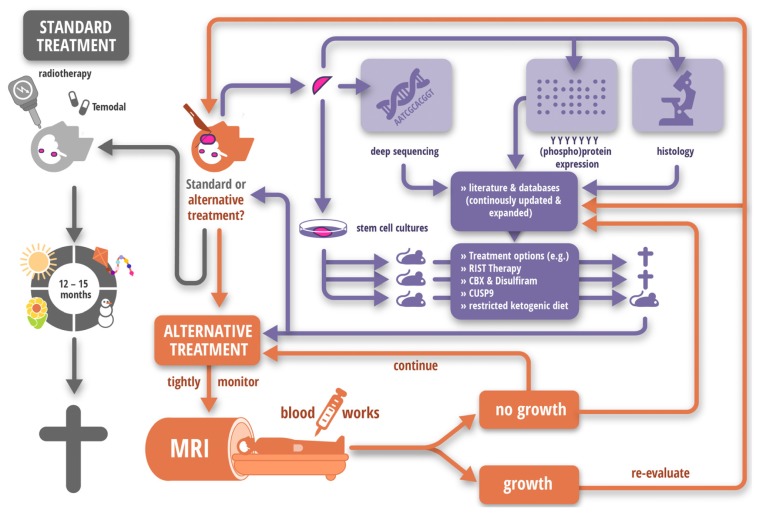
Example of a hypothetical treatment strategy for Glioblastoma. Glioblastoma is the most common primary brain tumor with an average patient’s life expectancy of approximately 12–15 month post-diagnosis [91,92]. Current standard of care is maximal surgical resection, followed by radiotherapy and several courses of chemotherapy in form of Temodal [91], as shown on the left. However, another future possibility could be to use the ever increasing information obtained by new sequencing techniques [93,94], proteomic profiling [95,96] and molecular histology [97] to devise an alternative treatment strategy. Of course, the feasibility and the risks would need to be discussed with the patient and discussions should be rendered on an individual basis. Furthermore, since culturing primary patient material as spheroids stably preserves the expression patterns [98] and these cells are also able to recapitulate the patients’ tumor in a murine environment [99], using a xenotransplant as a surrogate readout for patients’ responses to various treatment options could also be feasible. Combining these data sets, an alternative treatment strategy can then be devised. Important aspects that should be considered regarding the patient’s quality of life are: (1) Chemotherapeutic components should be included at metronomic doses to minimize adverse reactions/side effects; (2) The components should be easily administered to avoid repeated hospital stays, *i.e.*, oral administration is preferable to IV infusions. We feel that several approaches might fulfill these criteria with regards to glioblastoma, e.g., restricted ketogenic diet [100], CUSP9 [101], or our own approaches, the RIST protocol [86] or treatment with CBX [28] and Disulfiram [49]. The success of all of these approaches have been shown to varying degrees to be dependent on the AMAR, sequential dosing and chronification. Importantly, tumor progression needs to be tightly monitored, as well as patient’s general well-being, and should the tumor size not remain constant, therapy needs to be adapted accordingly. To avoid the emergence of therapy-resistant clones, consequently the treatment needs to be adapted when the tumor responds to treatment too well, and shrinks.

It is important to point out that we do not propose to chronify all tumors and avoid any attempt of cure. There are several types of cancer where the mortality rate decreased drastically over the last decades, for example breast, prostate colon and cervical malignancies, where we see a decrease in mortality from 1975 to 2010 between 30 and almost 60 percent [109]. However, survival rates for other tumors have remained unaltered or even worsened, for example glioblastoma or melanoma respectively [109,110]. Therefore, we believe that the next major challenge in apoptosis therapy is to identify those high risk patients, not just by cancer entity but also those which harbor a particular subtype of malignancy, that are best served by the treatment schedule proposed here (Figure 5).

## Figures and Tables

**Table 1.** AMAR in different malignancies. Instances of AMAR described in the literature, sorted by cancer cell type investigated. Of note, the repeated identification of the PI3K/Akt signaling network as AMAR mediator (see also Section II and Figure 3). Of additional interest is the frequent suggestion of integrin antagonists as potential therapy. Small molecule inhibitors are currently being clinically evaluated and show some promise in phase II trials [50–52]. Dark fields indicate lack of information. Abbreviations: **He**, Heterotypic cell-cell interaction; **ECM**, Cell-substrate interaction; **Ho**, Homotypic cell-cell interaction (modified and expanded from [40]).

Cancer	Adhesion type	Apoptosis inducing agents investigated	Molecular basis for AMAR	Therapeutic suggestion	Reference
Multiple myeloma	He, ECM	Melphalan	enhanced HSP70 expression	no concrete suggestion	[17]
Multiple myeloma	ECM	Doxorubicin, etoposide	not determined	no concrete suggestion	[23]
Multiple myeloma	ECM	CD95	increased soluble c-Flip	no concrete suggestion	[29]
Multiple myeloma	ECM	Doxorubicin, melphalan	not determined	no concrete suggestion	[27]
Multiple myeloma	ECM	Bortezomib	not determined	no concrete suggestion	[19]
Multiple myeloma	He	Doxorubicin	not determined	Integrin antagonists	[22]
Multiple myeloma	He, ECM	Lenalidomide	not determined	All-trans retinoic acid	[25]
Multiple myeloma	ECM	Doxorubicin, mitoxantrone	Up-regulation of p27Kip1	no concrete suggestion	[26]
Multiple myeloma	He	Melphalan, treosulfan, doxorubicin, dexamethasone, bortezomib	Rho/Rho-Kinase signaling	Statins	[13]
Multiple myeloma, acute myeloid leukemia	ECM	Tipifarnib	not determined	combine with bortezomib	[15]
Burkett’s lymphoma	ECM	Etoposide	Bcl-2 family	no concrete suggestion	[30]
B-cell lymphoma	He	Rituximab	not determined	Integrin antagonists	[21]
Histiocytic lymphoma	ECM	Mitroxantrone	not determined	no concrete suggestion	[14]
Lymphoma	He	Mitroxantrone	IAPs via NF-kappaB	no concrete suggestion	[31]
Lymphoblastic leukemia	He	Cytarabine, etoposide	not determined	no concrete suggestion	[32]
Chronic lymphocytic leukemia	He	Fludarabine	PI3K/Akt mediated signaling	PI3K inhibitor	[33]
Acute myeloid leukemia	ECM	Cytarabine	Bcl-2 via PI3K/Akt	Integrin antagonists	[16]
Acute myeloid leukemia	ECM	Daunorubicin	XIAP via PI3K/Akt	no concrete suggestion	[18]
Acute myeloid leukemia	ECM, He	Rutin, etoposide	GSK3beta	Rutin	[20]
Chronic myeloid leukemia	ECM	Melphalan, cytarabin, radiation	not determined	Integrin antagonists	[12]
Breast cancer	ECM	Paclitaxel, vincristine	PI3K/Akt mediated signaling	no concrete suggestion	[34]
Breast cancer	Ho	Staurosporine	Bcl-2 family	no concrete suggestion	[37]
Hepatocellular carcinoma	ECM	Paclitaxel	not determined	Integrin antagonists	[24]
Small cell lung cancer	ECM	Doxorubicin, etoposide	Tyrosine phosphorylation	Integrin antagonists	[35]
Glioblastoma	ECM	Etoposide	Bcl-2 family	no concrete suggestion	[36]
Glioblastoma	ECM	Radiation	not determined	no concrete suggestion	[38]
Glioblastoma	Ho, ECM	TRAIL	not determined	Carbenoxolone	[28]
